# CBARA1 Plays a Role in Stemness and Proliferation of Human Embryonic Stem Cells

**DOI:** 10.1371/journal.pone.0063653

**Published:** 2013-05-08

**Authors:** Kevin Chen, Lih-Tao Hsu, Cheng-Yi Wu, Shiun-Yin Chang, Hui-Ting Huang, Wannhsin Chen

**Affiliations:** Department of Tissue Regeneration Product Technology, Faculty of Biomedical Technology and Device Research, Industrial Technology Research Institute, Hsinchu, Taiwan; Baylor College of Medicine, United States of America

## Abstract

Human embryonic stem cells (hESCs) are capable of unlimited self-renewal and can generate almost all of the cells in the body. Although some pluripotency factors have been identified, much remains unclear regarding the molecules and mechanisms that regulate hESC self-renewal and pluripotency. In this study, we identified a mitochondrial gene, CBARA1, that is expressed in undifferentiated hESCs and that is down-regulated rapidly after cellular differentiation. To study its role in hESCs, endogenous CBARA1 expression was knocked down using shRNA. CBARA1 knockdown in hESCs resulted in down-regulation of Oct4 and Nanog expression, attenuated cell growth, and G0/G1 phase cell cycle arrest; however, knockdown did not noticeably affect apoptosis. Taken together, these results suggest that CBARA1 is a marker for undifferentiated hESCs that plays a role in maintaining stemness, cell cycle progression, and proliferation.

## Introduction

Human embryonic stem cells (hESCs) are pluripotent cells derived from the inner cell mass of blastocysts. Notably, hESCs can self-renew and differentiate into diverse specialized somatic cells [1], features that make hESCs a valuable tool to provide an unlimited supply of somatic cells for use in research, drug development, or regenerative medicine. Although under appropriate culture conditions hESCs can be maintained in an undifferentiated state over multiple passages, spontaneous differentiation inevitably occurs in culture. Accordingly, great effort has been made to identify the molecular mechanisms that regulate hESC self-renewal in order to improve the expansion of undifferentiated hESCs. hESCs express a set of transcription factors that are essential for the maintenance of hESC self-renewal and pluripotency, including Oct4, Nanog, and Sox2 [2]–[5]. These markers are frequently used to discriminate undifferentiated hESCs from differentiated cells in culture. Other factors and conditions have been identified that regulate pluripotency, such as cell adhesion molecules [6]–[8], growth factors [9]–[13], extracellular matrix [14], hypoxic culture [15], [16] and signaling pathway [17]–[19], and determination of their relevance in hESC self-renewal and pluripotency has helped improve hESC culture conditions. However, it is unknown whether other factors are also involved in regulating hESC self-renewal and pluripotency.

In a preliminary microarray study, we found that the MICU1 gene was present at higher levels in undifferentiated TW1 hESCs than in differentiated cells (data not shown). MICU1, also known as calcium-binding atopy-related autoantigen 1 (CBARA1), encodes a 54-kDa mitochondrial EF hand-motif containing protein that regulates calcium influx into mitochondria [20]. To date, the role of CBARA1 in hESCs has not been studied. Here we investigated the role of CBARA1 in hESC stemness, proliferation, cell cycle progression, differentiation, and apoptosis using short hairpin RNA (shRNA) to knockdown CBARA1 expression. Our findings demonstrate a role for CBARA1 in the regulation of hESC stemness, proliferation, and cell cycle progression.

## Materials and Methods

### hESC Maintenance and Differentiation

The hESC lines TW1, TW5 (ITRI and Lee Women’s Hospital, Taiwan) [21], H9 (WiCell Research Institute, Inc., Madison, WI) [1], and HES3 (ESI cell international, Singapore) [22] were maintained on feeder-free Stematrix® plates (Abnova, Taipei, Taiwan) in mouse embryonic fibroblast-conditioned medium (MEF-CM; Abnova) in an incubator at 37°C with 5% CO_2_. Culture medium was changed every 2 days, and cells were passaged weekly using manual dissection. All experiments with hESCs were conducted with prior approval from ITRI’s Institution Review Board. In some experiments, hESCs were treated with 100 ng/ml human recombinant noggin (Alpha Diagnostic International, San Antonio, TX) to induce differentiation as described by Gerrard et al. [23]. Spontaneous differentiation of hESCs was induced by growing them on Stematrix® in DMEM/F12 supplemented with 15% knockout serum replacement, 1% non-essential amino acids, 1 mM L-glutamine, and 0.1 mM β-mercaptoethanol (all from Invitrogen, Carlsbad, CA).

### Quantitative RT-PCR Analysis

RNA samples were extracted from cells using an RNeasy Mini Kit (Qiagen, Hilden, Germany) and reverse transcribed to cDNA using the High Capacity cDNA Reverse Transcription Kit (Applied Biosystems, Foster City, CA). Quantitative RT-PCR (qRT-PCR) analyses were performed using Taqman Gene Expression Assays (Applied Biosystems) on an ABI StepOne Plus Real-Time PCR System (Applied Biosystems) according to the manufacturer’s instructions. The expression of each gene was analyzed in triplicate under the following PCR conditions: 95°C for 20 seconds, 40 cycles at 95°C for 1 second, and 60°C for 20 seconds. Results were normalized using the housekeeping gene GAPDH and analyzed via the △△Ct method using StepOne™ software version 2.2.2 (Applied Biosystems). FAM-labeled primers for qRT-PCR (Applied Biosystems) were as follows: CBARA1 (Hs00246104_m1), Oct4 (Hs00999632), Nanog (Hs04260366_q1), Pax6 (Hs00240871_M1), and GAPDH (Hs99999905_m1).

### Western Blot Analysis

Cells were lysed by mixing them with radio-immunoprecipitation assay buffer (10 mM Na_2_HPO_4_, 150 mM NaCl, 1 mM EDTA, 1% NP40, 0.1% SDS, and 1% sodium deoxycholate) containing protease inhibitor cocktail (Roche Molecular Diagnostics, Mannheim, Germany) and phosphatase inhibitors (Sigma, St Louis, MO). Protein concentration in the cell lysates were quantified using a Bradford protein assay kit (Bio-Rad, Hercules, CA) according to the manufacturer’s instructions. For Western blot analysis, the prepared samples were electrophoresed on 12% SDS-polyacrylamide gel under reducing conditions. After electrophoresis, the proteins were transferred to PVDF membrane using the iBlot™ Dry Blotting System (program P3 at 7 minutes; Invitrogen). The membrane was blocked with 5% skim milk for 1 hour at room temperature followed by incubation with mouse anti-CBARA1 (1∶1000; Abnova), mouse anti-Oct4 (1∶1000; Millipore, Temecula, CA), or mouse anti-α tubulin (1∶10000; AbFrontier, Seoul, South Korea) at 4°C overnight. The membrane was washed 3 times for 5 minutes each with Tris-buffered saline and then incubated with secondary antibody for 1 hour at room temperature. The proteins were visualized using a SuperSignal West Femto Chemiluminescent Substrate kit (Thermo Scientific, Rockford, IL) and quantified by densitometric analysis using FluorChemSP software with α-tubulin as a reference.

### Immunocytochemistry and Flow Cytometric Analyses

Immunostaining and flow cytometric analyses were performed on cells as previously described [21] using the following antibodies: rabbit anti-Oct4 (1∶200; Abcam), mouse anti-CBARA1 (1∶100; Abnova), rabbit anti-IgG (1∶1000; Jackson Laboratories, Bar Harbor, ME), and mouse anti-IgG (1∶1000; Jackson Laboratories). Secondary antibodies used were goat anti-mouse Alexa 594 and goat anti-rabbit Alexa 488. DAPI nuclear counterstain (1∶10 000; 5 mg/ml, Roche Molecular Diagnostics) was used to label cell nuclei. Fluorescently labeled cells were imaged using an inverted fluorescence microscope (Carl Zeiss MicroImaging, Jena, Germany). Flow cytometric analysis was performed using a BD FACSCalibur flow cytometer, and data were analyzed using CellQuest software (Becton Dickinson, Franklin Lakes, NJ).

### shRNA-mediated Knockdown of CBARA1

MISSION® shRNA lentiviral vectors (Sigma) were used to knock down the expression of CBARA1 in hESCs. The following shRNA lentiviral particles were used: non-target control shRNA (SHC002V) and CBARA1 shRNA (TRCN0000053372). The lentiviral transduction was performed according to the manufacturer’s instructions with slight modifications. Briefly, HES3 cells or H9 hESCs (1×10^5^) were transduced with lentiviral particles at an MOI of 20 in MEF-CM containing 6 µg/ml polybrene (Sigma) at 37°C with 5% CO_2_. After incubation for 24 hours, the medium was changed and the transduced cells were cultured in MEF-CM for 4 additional days before puromycin selection (1 µg/ml; Sigma). The efficiency of CBARA1 knockdown in hESCs was determined by qRT-PCR and flow cytometric analysis 7 days after puromycin selection.

### Cell Proliferation and Cell Cycle Analyses

To determine the effect of CBARA1 on hESC proliferation, the total number of shRNA-transduced cells was quantified using a trypan blue exclusion assay on days 1, 3, and 5 after seeding cells at an initial cell density of 4×10^4^ cells/well. For cell cycle analysis, cells were stained with propidium iodide (PI, 50 µg/ml; Sigma) and analyzed using flow cytometry. Data was acquired using CellQuest software (Becton Dickinson), and the percentages of G0/G1, S, and G2/M-phase cells were calculated using the MODFIT-LT software (Verity Software House, Topsham, ME).

### Annexin V Apoptosis Analysis

To detect early cell apoptosis, shRNA-transduced or non-transduced hESCs were analyzed using a Molecular Probes Vybrant® Apoptosis Assay Kit (Invitrogen) according to the manufacturer’s instructions. Annexin V-Alexa 488 and PI-stained cells were analyzed using flow cytometry.

### Statistical Analysis

Data are presented as mean ± standard deviation. A two-tailed unpaired student’s *t*-test was used for most comparisons using SigmaPlot version 10.0 (Systat Software Inc., San Jose, CA). Statistical significance was established at *P*<0.05.

## Results

### CBARA1 is a Marker for Undifferentiated hESCs

**Figure 1:**
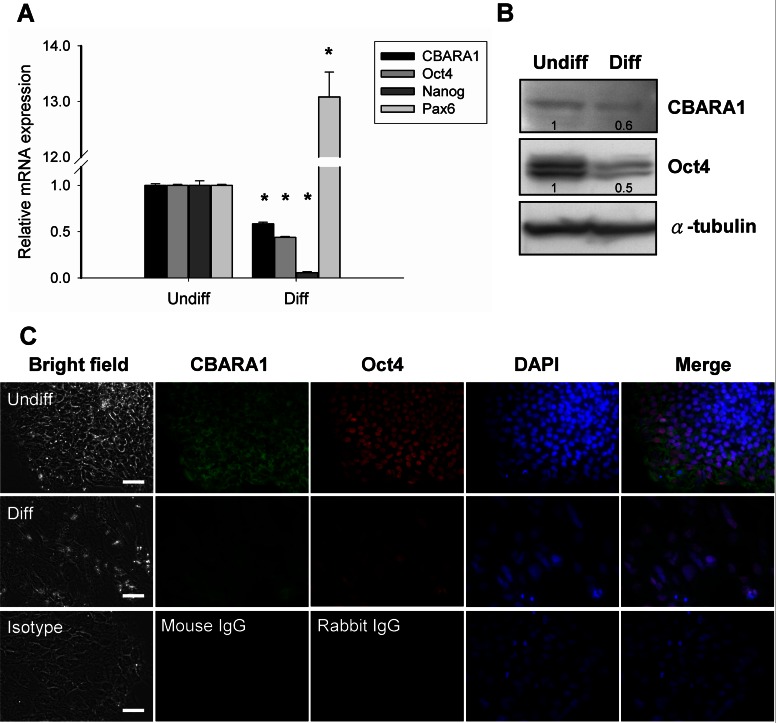
CBARA1 is a marker for undifferentiated hESCs. (A) Quantitative PCR was used to determine CBARA1 and Oct4 mRNA levels in undifferentiated TW1 hESCs and noggin-induced differentiated cells. Gene expression was normalized to GAPDH expression and is presented as the fold-change compared to expression in undifferentiated hESCs. The bars show the means ± standard deviations of three independent experiments. *Significant difference compared to undifferentiated hESCs (*P*<0.01). (B) Western blot showing CBARA1 protein levels in undifferentiated and 7-day differentiated hESCs. α-tubulin was used as a loading control. The relative reduction of CBARA1 in 7-day differentiated cells was quantified by densitometry as shown under the blots. (C) Representative images show CBARA1 and Oct4 immunostaining in undifferentiated or 7-day differentiated hESCs. Abbreviations: Undiff, undifferentiated hESC; Diff, differentiated hESC. Scale bar = 50 µm.

To determine whether CBARA1 is enriched in undifferentiated hESCs compared to differentiated cells, we analyzed the mRNA expression levels of CBARA1 in undifferentiated and noggin-induced differentiated hESCs using quantitative RT-PCR. CBARA1 mRNA was expressed at high levels in undifferentiated hESCs but was markedly down-regulated after differentiation (Figure 1A). In 7-day differentiated cells, CBARA1 expression was down-regulated to a level that was 58% of the level in undifferentiated hESCs. Noggin-induced hESC differentiation also resulted in Oct4 and Nanog down-regulation and in up-regulation of Pax6, an early neuroectodermal marker (Figure 1A). Next, we evaluated CBARA1 expression in hESCs at the protein level by Western blot analysis. The CBARA1 protein was detected as a ∼50 kDa band in undifferentiated hESCs (Figure 1B), which is similar to the predicted molecular size of CBARA1. This band was reduced in intensity in differentiated cells (Figure 1B). Notably, an additional immunoreactive band was observed at ∼40 kDa (data not shown). The Oct4 protein was also down-regulated in differentiated cells (Figure 1B). Immunocytochemistry showed that CBARA1 expression was localized predominantly to the cytoplasm, and that the protein was co-expressed with Oct4 in undifferentiated hESCs (Figure 1C). However, expression of both CBARA1 and Oct4 was reduced in 7-day differentiated cells (Figure 1C). These results indicated that at both the mRNA and protein levels, the expression of CBARA1 positively correlated with the hESC differentiation state, identifying it as a potential marker for undifferentiated hESCs.

**Figure 2:**
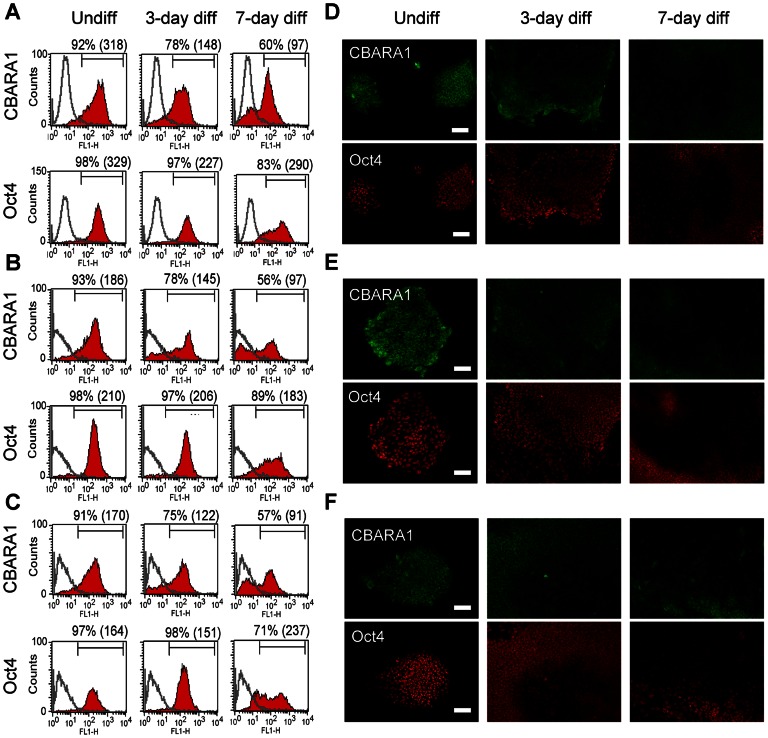
Expression of CBARA1 in hESCs following spontaneous differentiation. (A–C) Flow cytometry shows the percentage and mean fluorescent intensity (in brackets) of CBARA1- or Oct4-positive cells in undifferentiated, 3-day, or 7-day differentiated cells. (D–E) Representative images showing CBARA1 and Oct4 immunostaining in undifferentiated and in 3- or 7-day spontaneously differentiated cells. One graph or image that was representative of three independent experiments is shown for the TW1 (A, D), HES3 (B, E), and TW5 (C, F) hESC lines. Scale bar = 100 µm.

Since spontaneous differentiation occurs readily in hESC culture, we used flow cytometry and immunocytochemistry to analyze CBARA1 and Oct4 expression in TW1 hESCs following spontaneous differentiation. Spontaneous differentiation of hESCs was induced by growth factor withdrawal. Before differentiation, over 90% of the cells were positive for both CBARA1 and Oct4 (Figure 2A). Following spontaneous differentiation, the percentage of CBARA1-postive cells rapidly decreased compared to the percentage of Oct4-positive cells (Figure 2A). The percentage of CBARA1-positive cells decreased to ∼78% of the original level on day 3 and decreased further to ∼60% of the original level on day 7 of differentiation (Figure 2A). In contrast, the percentage of Oct4-positive cells remained relatively constant, showing expression that was >90% of the original level on day 3 and ∼83% of the original on day 7 of differentiation (Figure 2A). Moreover, the mean fluorescent intensity of CBARA1 expression was reduced to a greater extent over time than was Oct4 expression in hESCs after differentiation (Figure 2A). Consistent with the flow cytometry data, immunocytochemistry demonstrated that CBARA1 was down-regulated more rapidly than Oct4 in hESCs in response to spontaneous differentiation (Figure 2D). Taken together, these results suggest that CBARA1 is a sensitive marker for detecting hESC spontaneous differentiation. Similar results were obtained in the HES3 and TW5 hESC lines (Figure 2B–C, E–F).

### CBARA1 Plays a Role in hESC Stemness and Proliferation

**Figure 3:**
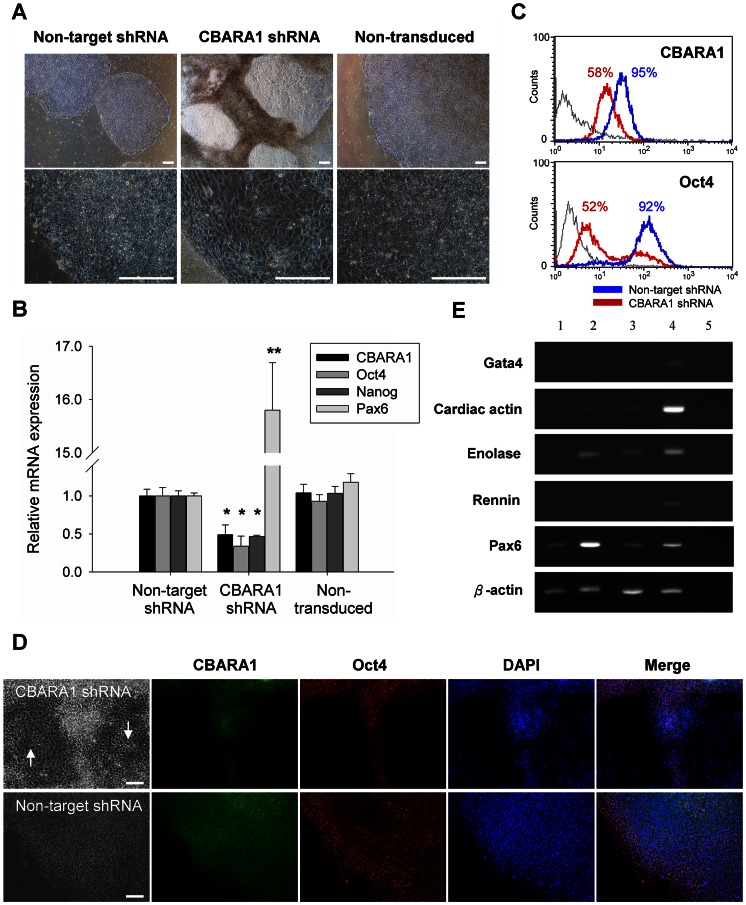
Effect of CBARA1 on hESC stemness. (A) Representative phase-contrast images show CBARA1 shRNA vector-transduced, non-target shRNA vector-transduced, and non-transduced hES3 cells. (B–C) Quantitative PCR and flow cytometry show reduced CBARA1 and Oct4 mRNA and protein expression in CBARA1 shRNA vector-transduced hESCs compared to non-target shRNA vector-transduced and non-transduced controls. Gene expression was normalized to that of GAPDH and is presented as fold-change compared to the non-target shRNA control. Bars represent the means ± standard deviations of three independent experiments. *Difference compared to non-target shRNA control (*P*<0.05). **Difference compared to non-target shRNA control (*P*<0.01) (D) Representative images show CBARA1 and Oct4 immunostaining in non-target shRNA vector-transduced, CBARA1 shRNA vector-transduced, and non-transduced hESCs. Regions of cell differentiation are indicated with arrows. (E) RT-PCR shows the expression of specific markers for the three germ layers in non-transduced hESCs and in hESCs transduced with CBARA1 shRNA or non-target shRNA vectors. Lane 1, HES3 non-target shRNA; lane2, HES3 CBARA1 shRNA; lane 3, H9 non-target shRNA; lane4, H9 CBARA1 shRNA; lane5, negative control. Scale bar = 100 µm.

To study the role of CBARA1 in hESCs, we knocked down its expression using shRNA. hESCs transduced with CBARA1 shRNA or non-target shRNA were selected with puromycin and maintained in feeder-free culture conditions for 7 days. Expression of CBARA1 protein and mRNA was reduced more than 40% in CBARA1 shRNA-transduced cells (Figure 3B–C). In contrast, CBARA1 expression was not significantly reduced in cells transduced with non-target shRNA compared with non-transduced hESCs (Figure 3B). Strikingly, CBARA1 knockdown in hESCs induced morphological differentiation that was coincident with down-regulation of Oct4 and Nanog expression (Figure 3A-D). CBARA1-negative and Oct4-negative cells exhibited epithelial-like morphology with large nuclei and abundant cytoplasm (Figure 3D). Since CBARA1 shRNA induced hESC differentiation, we sought to determine what lineage of differentiated cells was generated by CBARA1 knockdown. RT-PCR analysis demonstrated that CBARA1 knockdown in H9 hESCs led to the formation of all three germ layers: ectoderm (pax6), mesoderm (enolase, rennin), and endoderm (gata4, cardiac actin). However, HES3 cells did not show endodermal differentiation following CBARA1 knockdown. This difference in H9 and HES3 cells might be due to genetic differences in the two cell lines. Overall, these findings suggest that CBARA1 down-regulation induces differentiation that does not result in a specific cell lineage (Figure 3E).

**Figure 4:**
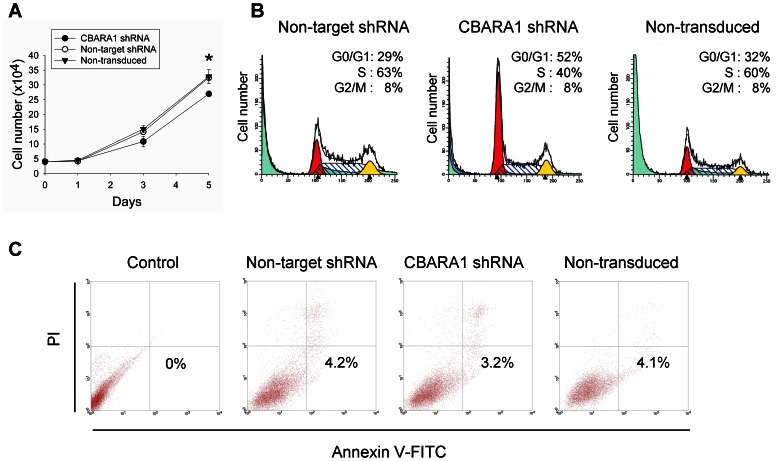
Effect of CBARA1 on hESC proliferation and apoptosis. (A) Graphs show a reduced cell proliferation in CBARA1 shRNA vector-transduced hES3 cells for up to 5 days after puromycin selection compared to the non-target shRNA control. Statistical analysis was performed using the Student’s t-test. *Difference compared to non-target shRNA control (*P*<0.01). (B) Cell cycle analysis shows the cell cycle distribution of non-target shRNA vector-transduced, CBARA1 shRNA vector-transduced, and non-transduced hESCs at the G0/G1, S, and G2/M phases. One graph is shown representative of two independent experiments. The percentage of cells in each phase of the cell cycle was calculated using MODFIT-LT software. (C) Annexin V/PI flow cytometry analysis showing the percentage of early apoptotic cells (Annexin V+/PI- in the lower right quadrant) in CBARA1 shRNA-treated, non-target shRNA-treated, non-transduced, and unstained control hESCs. One graph is shown that is representative of two independent experiments. Abbreviations: PI, propidium iodide.

To examine the effect of CBARA1 knockdown on hESC proliferation, we counted the total number of shRNA-transduced cells maintained under pluripotent culture conditions for up to 5 days after puromycin selection. As shown in Figure 4A, the proliferative ability of the CBARA1-knockdown hESCs was significantly reduced compared to the non-target shRNA control cells over the test period, suggesting that reducing the expression of CBARA1 attenuated hESC proliferation in vitro. Next, we analyzed the cell cycle distribution of CBARA1-knockdown hESCs using flow cytometry. The percentage of cells in the G0/G1 phases in CBARA1 knockdown cells, ∼52%, was higher than the percentage of non-target transduced cells, ∼29%, or non-transduced cells, ∼32% (Figure 4B). In addition, fewer of the CBARA1-knockdown hESCs were in S-phase (∼40%) as compared with the non-target shRNA transduced cells (∼63%) (Figure 4B). These results suggest that CBARA1 knockdown blocks cell cycle progression in hESCs. To determine whether CBARA1 is associated with apoptosis in hESCs, we performed the Annexin V/propidium iodide assay to detect apoptosis in CBARA1-knockdown cells and control cells. About 3% of the CBARA1 knockdown hESCs were apoptotic, which was not much different than the ∼4% of non-target shRNA control cells. This indicated that CBARA1 knockdown did not affect apoptosis (Figure 4C). Collectively, these results suggest that CBARA1 acts to sustain the undifferentiated proliferation of hESCs and facilitates cell cycle progression rather than inhibiting apoptosis.

## Discussion

In the present study, we characterized the expression and investigated the function of a recently identified gene, CBARA1, in hESCs. We found that CBARA1 was expressed in undifferentiated hESCs and was down-regulated markedly following differentiation. This change in expression was similar to that seen for Oct4, a transcription factor that is involved in the self-renewal of undifferentiated hESCs. Furthermore, suppression of CBARA1 expression in hESCs using shRNA led to cell differentiation, decreased cell proliferation, and aberrant cell cycle progression. These findings suggest that CBARA1 can be used as a marker for undifferentiated hESCs that would distinguish them from their early-differentiated derivatives and that CBARA1 plays a role in maintaining hESC stemness and proliferation.

Spontaneous differentiation of hESCs occurs frequently in culture. Thus, it is important to identify sensitive markers that can rapidly and accurately assess the differentiation state of the hESCs. Although pluripotency markers, including Oct4 and Nanog, are essential for hESC pluripotency, they are not sensitive enough to detect early changes in undifferentiated hESC cultures because their expression is down-regulated very slowly as the hESCs differentiate [24], [25]. In this study, we showed that CBARA1 expression declines earlier than Oct4 expression following spontaneous differentiation. Specifically, changes in Oct4 protein expression were not observed until day 7 after differentiation, while changes in CBARA1 were notable by day 3. Furthermore, we demonstrated that CBARA1 knockdown leads to more rapid down-regulation of Oct4 in hESCs. These findings suggest that CBARA1 may be required to maintain Oct4 expression, which may account for the faster kinetics of CBARA1 down-regulation at the early stage of cell differentiation. Other hESC pluripotency-associated markers, including UTF-1 and Lefty A, are also down-regulated early in hESC differentiation [26], [27]. In combination with these markers, CBARA1 may help reliably assess the differentiation state of hESCs.

The mechanisms underlying the maintenance of hESC self-renewal and pluripotency are complex and remain unclear. Recently, it has become increasingly evident that mitochondrial function plays an important role in controlling embryonic stem cell proliferation and pluripotency [28]–[31]. For example, Mandal et al. [28] showed that disruption of mitochondrial function in mouse embryonic stem cells (mESCs) and hESCs using the protonophore carbonyl cyanide m-chlorophenylhydrazone results in reduced proliferation and compromised differentiation potential. Varum et al. [32] showed that treating mESCs or hESCs with the mitochondrial electron transport inhibitor antimycin A causes increased expression of Nanog and reduces the expression of genes associated with differentiation. In addition to mitochondrial function, other groups have demonstrated the involvement of specific mitochondrial proteins in maintaining ES cell pluripotency and differentiation [33], [34]. Growth factor erv1-like (Gfer), a FAD-dependent sulfhydryl oxidase that predominantly localizes to the intermembrane space of mitochondria, maintains the pluripotency of mESCs by modulating dynamin-related protein 1 expression. The disruption of Gfer in mESCs leads to decreased expression of the pluripotency markers Nanog, Oct4, and SSEA1, to reduced survival, and to impaired embryoid body formation [33]. In mice, targeted deletion of Ptpmt1 (a mitochondrial phosphatase localized exclusively to the mitochondrial inner membrane) results in postimplantation lethality and in impaired growth of the inner cell mass of blastocysts and also prevents the derivation of ES clones [34]. Ptpmt1-depleted ES cells exhibit decreased growth and loss of differentiation capabilities. These studies suggest that the mitochondrion is a key regulator of proliferation and differentiation in pluripotent stem cells.

In line with these previous studies, we showed that the mitochondrial protein CBARA1 is also involved in the control of hESC proliferation and differentiation. shRNA-mediated knockdown of CBARA1 in hESCs reduced cell proliferation, arrested cells at the G0/G1 phase, and down-regulated expression of the pluripotency marker Oct4. As CBARA1 is a key regulator of mitochondrial Ca^2+^ uptake [20], we speculate that the effect of CBARA1 on hESC self-renewal might be mediated by changes in calcium homeostasis. This hypothesis is in accordance with the findings of Nunez et al. [35], who showed that salicylate-induced inhibition of mitochondrial Ca^2+^ uptake leads to inhibition of store-operated Ca^2+^ entry (SOCE) and impairs cell proliferation in Jurkat and human colon cancer cells. In this context, it is interesting to note that a recent study demonstrated that functional SOCE is present in mESCs and that prevention of SOCE attenuates mESC proliferation, arrests cells at the G0/G1 phase, and decreases the expression of pluripotent markers [36]. Conversely, stimulation of SOCE using 17β-estradiol increases mESC proliferation [36]. Thus, we hypothesize that CBARA1 maintains hESC proliferation and pluripotency by sustaining SOCE through activation of mitochondrial clearance of the entering Ca^2+^. Since SOCE is an early event in signaling transduction, it is likely that multiple downstream transducing proteins could be recruited in the proliferative or pluripotency-associated signaling pathway. Further work is required to identify the exact mechanism(s) involved.

Although CBARA1 is expressed in undifferentiated hESCs, it is also expressed in other cell types, such as epidermal keratinocytes, dermal endothelial cells, and HeLa cells; further, it is expressed at high levels in multiple tissues included skeletal muscle, kidney, lung, and spleen [20], [37], [38]. Similarly, other pluripotency-associated mitochondrial proteins, such as Gfer and Ptpmt1, are also expressed in mouse embryonic fibroblasts [33], [34]. Interestingly, these mitochondrial proteins seem to play more important roles in ES cells than in differentiated cells, since ES cells respond more dramatically than fibroblasts to mitochondrial dysfunction induced by pepmt1 or Gfer deficiency. In this regard, it is possible that CBARA1 regulation of hESC proliferation is cell-type specific and that it may have divergent effects in other cells.

In conclusion, these experiments demonstrated that CBARA1 is a marker of undifferentiated hESCs that is down-regulated rapidly in response to differentiation. We showed that knockdown of CBARA1 expression in hESCs results in a loss of stemness, reduced cell proliferation, and arrested cell cycle progression. The effects of CBARA1 expression in hESCs observed in this study support the notion that mitochondria play a role in regulating hESC stemness and self-renewal. These findings warrant further studies to investigate whether mouse CBARA1 plays similar roles in mouse ES cells.
